# Discovering the effect of combination of celecoxib and sorafenib on hepatocellular carcinoma

**DOI:** 10.1007/s12672-024-01203-w

**Published:** 2024-07-31

**Authors:** Wang Gu, Dongyun Zeng, Chao Zhang

**Affiliations:** 1 Hepatological Surgery Department, The First Affiliated Hospital of Anhui Medical University, 218 Jixi Road, Shushan District, Hefei City, 230032 Anhui Province China; 2 Clinicopathological Diagnosis and Research Center, The Affiliated Hospital of Youjiang Medical University for Nationalities, Baise, China; 3 Key Laboratory of Tumor Molecular Pathology of Guangxi Higher Education Institutes, Baise, China

**Keywords:** Celecoxib, Hepatocellular carcinoma, Differentially expressed genes, Immune infiltration, Bioinformatics, Key genes, GEO database

## Abstract

**Introduction:**

Hepatocellular carcinoma (HCC) is a common and fatal cancer, and its molecular mechanisms are still not fully understood. This study aimed to explore the potential molecular mechanisms and immune infiltration characteristics of celecoxib combined with sorafenib in the treatment of HCC by analyzing the differentially expressed genes (DEGs) from the GSE45340 dataset in the GEO database and identifying key genes.

**Methods:**

The GSE45340 dataset was downloaded from the GEO database, and DEGs were screened using GEO2R, and visualization and statistical analysis were performed. Metascape was used to perform functional annotation and protein–protein interaction network analysis of DEGs. The immune infiltration was analyzed using the TIMER database, and the expression of key genes and their relationship with patient survival were analyzed and verified using the UALCAN database.

**Results:**

A total of 2181 DEGs were screened through GEO2R analysis, and heat maps were drawn for the 50 genes with the highest expression. Metascape was used for enrichment analysis, and the enrichment results of KEGG and GO and the PPI network were obtained, and 44 core genes were screened. Analysis of the TIMER database found that 12 genes were closely related to tumor immune infiltration. UALCAN analysis further verified the differential expression of these genes in HCC and was closely related to the overall survival of patients.

**Conclusions:**

Through comprehensive bioinformatics analysis, this study identified a group of key genes related to the treatment of HCC with celecoxib combined with sorafenib. These genes play an important role in tumor immune infiltration and patient survival, providing important clues for further studying the molecular mechanism of HCC and developing potential therapeutic targets.

## Introduction

Hepatocellular carcinoma (HCC) is a type of primary cancer. It ranks fifth among the most common cancers in the world and third among the most fatal cancers [1]. Hepatocellular carcinoma is the third leading cause of cancer deaths worldwide, with a relative 5-year survival rate of approximately 18%. The similarity between incidence and mortality (830,000 deaths per year) underlines the dismal prognosis associated with this disease [2]. The anticancer effect of kinase inhibitors (such as sorafenib) in preclinical research shows that it has a good prospect, and has been widely used in clinical hepatocyte therapy. These inhibitors mainly work by blocking the mitogen activated protein kinase / extracellular signal regulated kinase (ERK) pathway. This pathway is up-regulated in different types of cancer during tumor progression, which is closely related to the occurrence and development of liver cancer. However, many patients who initially respond to the treatment targeting the ERK pathway will develop drug resistance later, resulting in poor treatment outcomes [3–5].

Celecoxib has a variety of pharmacological activities and functions, among which the most surprising is that celecoxib can be involved in the treatment of cancer [6]. Cancer microenvironment is often accompanied by high expression of various molecules, such as inflammatory mediators such as arachidonic acid and cytokines [7]. In cancer cells, cyclooxygenase-2 pathway is up-regulated, resulting in an excessive increase in prostaglandin production. This leads to the proliferation of tumor cells. Many cancer treatments have been found to inhibit cyclooxygenase-2. Inhibition of cyclooxygenase-2-mediated prostaglandin excess leads to abnormal cell growth control. Cyclooxygenase-2 inhibitors act by inhibiting the overexpression of prostaglandins and act as chemopreventive agents. In animal experiments of colorectal cancer [8], lung cancer [9] and breast cancer [10], it is found that blocking COX-2 pathway can effectively prevent tumor growth and metastasis.

Studies have shown that cyclooxygenase-2 (COX-2) plays a role in the occurrence and development of hepatocellular carcinoma, and selective COX-2 inhibitors (COX-2 inhibitors) have obvious anti proliferative and pro apoptotic effects in human HCC cell lines [11–13]. Several genes involved in the regulation of apoptosis, ER stress response, DNA damage response, cell proliferation, and invasion (including BIRC5, Hrk, DDIT3/CHOP, TRB3, CCND1, MT2A, LARP6, YAP1, FABP1, and DKK1) were reported to be affected when CLX and SOR were applied alone [14, 15]. These genes have now been shown to have a synergistic regulatory effect when treated in combination, suggesting that they may play a role in enhancing anti-tumor effects when cells receive SOR + CLX combination therapy [16]. There are also relevant studies showing that celecoxib can enhance the efficacy of sorafenib [16], but its specific mechanism is not clear. This study aims to analyze the effect of celecoxib combined with sorafenib on gene expression of hepatoma cells by bioinformatics, and then speculate the possible mechanism and pathway related to its effect.

## Methods and materials

### Obtaining data from GEO database

The dataset of related studies was retrieved from the GEO database, and the GSE45340 dataset was downloaded. The platform used was the Agilent-014850 whole human genome microarray 4 × 44 k G4112F (feature number version). The data set contained four samples (GSM1102674: HepG2 cells_ SOR + CELE treated_ 48 h vs untreated HepG2_ rep1, GSM1102675: HepG2 cells_ SOR + CELE treated_ 48 h vs untreated HepG2_ rep2 (dye swap), GSM1102676: Huh7 cells_ SOR + CELE treated_ 48 h vs untreated HepG2 cells_ rep1, GSM1102677: Huh7 cells_ SOR + CELE treated_ 48 h vs untreated HepG2 cells_ Rep2 (dye swap)).

### Identification of differentially expressed genes

Use GEO2R to screen the differentially expressed genes (DEGs), and then further visualize and statistically analyze the DEGs. For the GEO2R analysis, default parameters were utilized, including a two-sided Student’s t-test to identify differentially expressed genes (DEGs) between the treatment and control groups. The p-value cutoff and log2 fold change threshold were set at default values, with significant DEGs determined based on statistical significance (p < 0.05) and fold change (> 2 or < − 2). The default settings were chosen to maintain consistency with established practices and to facilitate comparison with other studies.

### Functional annotation and protein–protein interaction network of the DEGs

Metascape [17] (http://metascape.org/gp/index.html#/main/step1) is a simple and powerful tool for gene function annotation analysis, which can help users apply the current popular bioinformatics analysis methods to batch gene and protein analysis, so as to realize the cognition of gene or protein function. Metascape not only includes the enrichment analysis of biological pathways, the analysis of protein–protein interaction network structure and abundant gene annotation functions, but also presents the results in a high-quality graphic language that biologists can easily understand. So as to screen out the hub genes and related enrichment pathways.

### Immunoinfiltration analysis

The TIMER (Tumor Immune Estimation Resource) database is a tool for tumor immune infiltration analysis, which can help researchers understand the abundance and activity of different immune cells in the tumor microenvironment. First, open the TIMER database website (https://cistrome.shinyapps.io/timer/). On the homepage of the website, select the hepatocellular carcinoma tumor type. The TIMER database covers a variety of tumor types, including common cancer types. Immune infiltrates were selected for analysis. The TIMER database provides a variety of different analysis options, including gene expression, survival analysis, immune cell abundance, and more. Enter a gene or gene set and click “Analyze” or a similar button to run the selected analysis. After the analysis is completed, the TIMER database will provide corresponding results. These results may include graphs of immune cell abundance.

### Analysis and screening of key genes with UALCAN

UALCAN [18] is a comprehensive, user-friendly, interactive web resource for analyzing cancer data. It is built on Perl CGI, with high-quality graphics using JavaScript and CSS. UALCAN design, provides convenient access to open cancer histochemical data (TCGA, met500 and cptac), (b) allows users to identify biomarkers or perform potentially interested genes verified in silicon, (c) provide graphic and plot descriptions as expression profiles of encoded proteins and patient survival information, miRNA coding and lincrna coding genes, (d) epigenetic regulation of gene expression evaluation by promoter methylation, (e) For the analysis of Pan oncogene expression, (f) provide more information about the selected gene / target by connecting HPRD, genecards, PubMed, targetscan, the human protein atlas, drugbank, open targets and GTEX. These resources enable researchers to collect valuable information and data on genes / objectives of interest.

### Statistical analysis

The statistical software R version 4.2.0 was applied to perform the statistical analysis, with p-values of < 0.05 suggesting statistical significance.

## Result

### Acquisition of DEGs

**Fig. 1:**
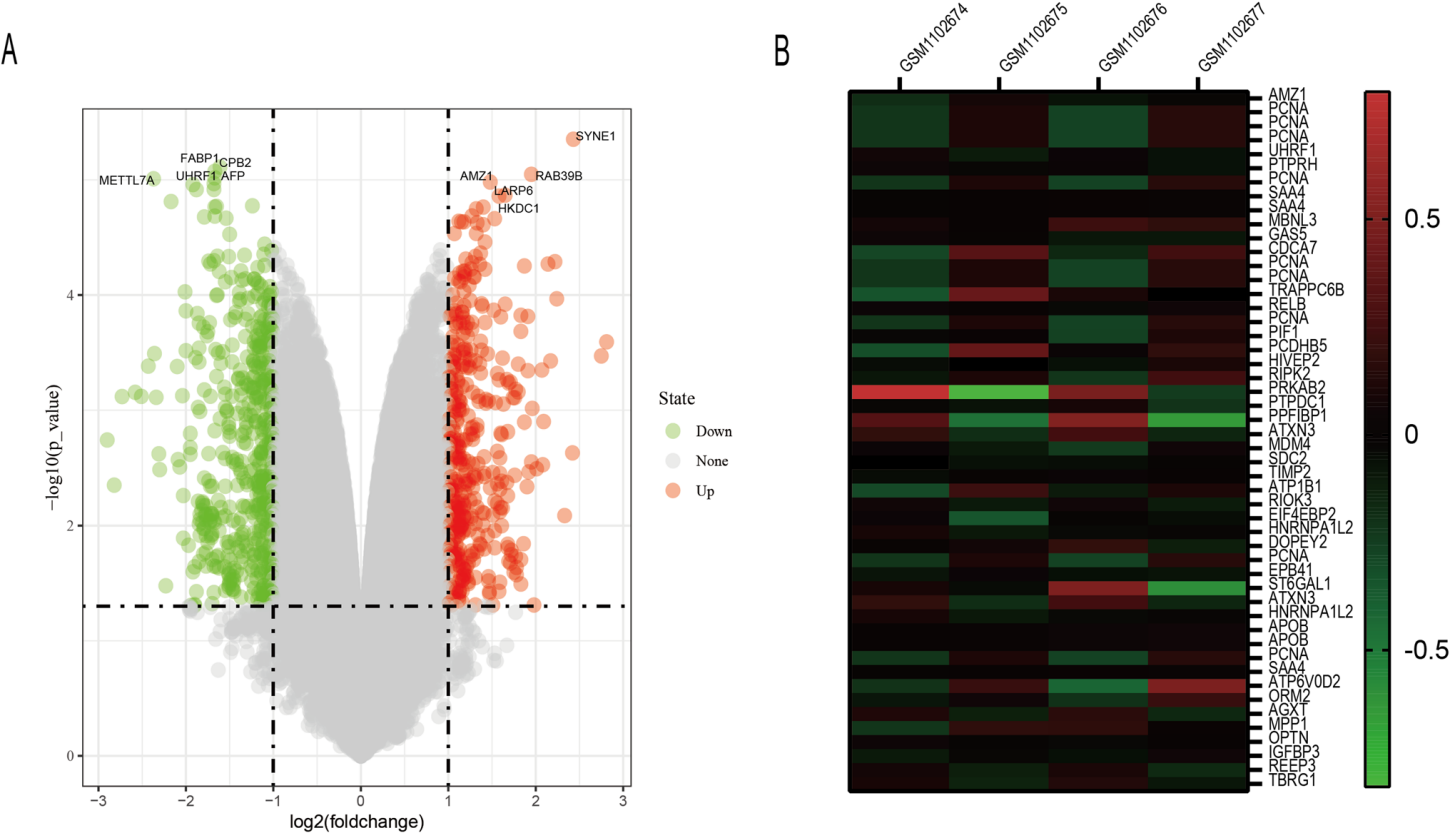
**A** Volcano map made by 2181 differential genes, **B** The expression heat map of the 50 genes with the most significant difference

Through GEO2r analysis of the untreated group and the combination group, we got the differentially expressed genes, and drew the volcano map (Fig. 1A). Further screening of the GEO2R analysis results identified 2181 differentially expressed genes. The 50 genes with the highest expression difference were selected. Figure 1B shows these 50 differential genes.

### Analysis of DEGs in metascape

**Fig. 2:**
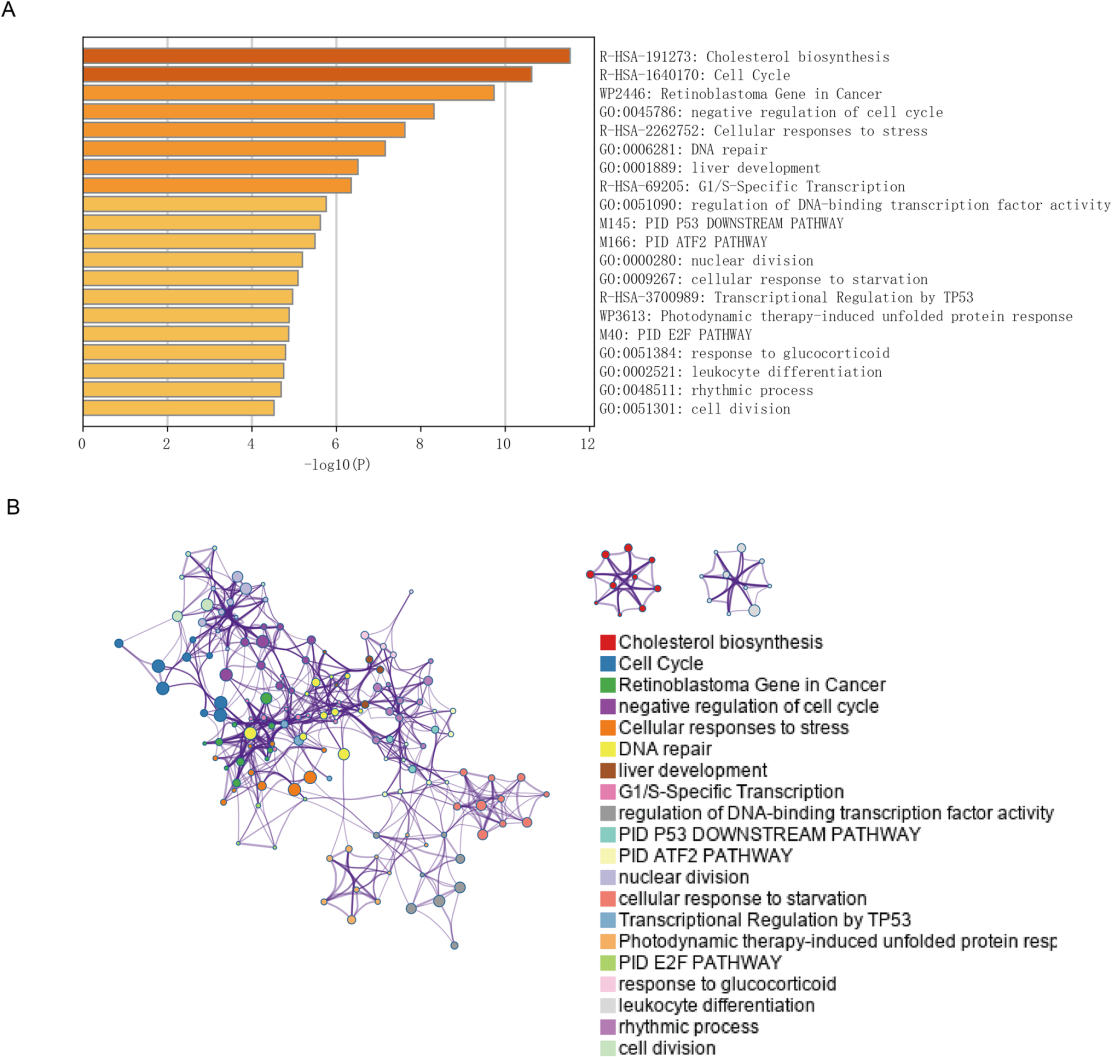
**A** The enrichment analysis results of the top 50 differential genes include go and KEGG, **B** Enrichment analysis pathway network diagram of the top 50 differential genes

Metascape is a software for enrichment analysis. We uploaded the differential genes obtained in the above steps to this software for analysis, and obtained the enrichment analysis results of KEGG and GO (Fig. 2A). Network is a very important means of expression. The results of enrichment analysis can be further analyzed to obtain the correlation between them (Fig. 2B).

**Fig. 3:**
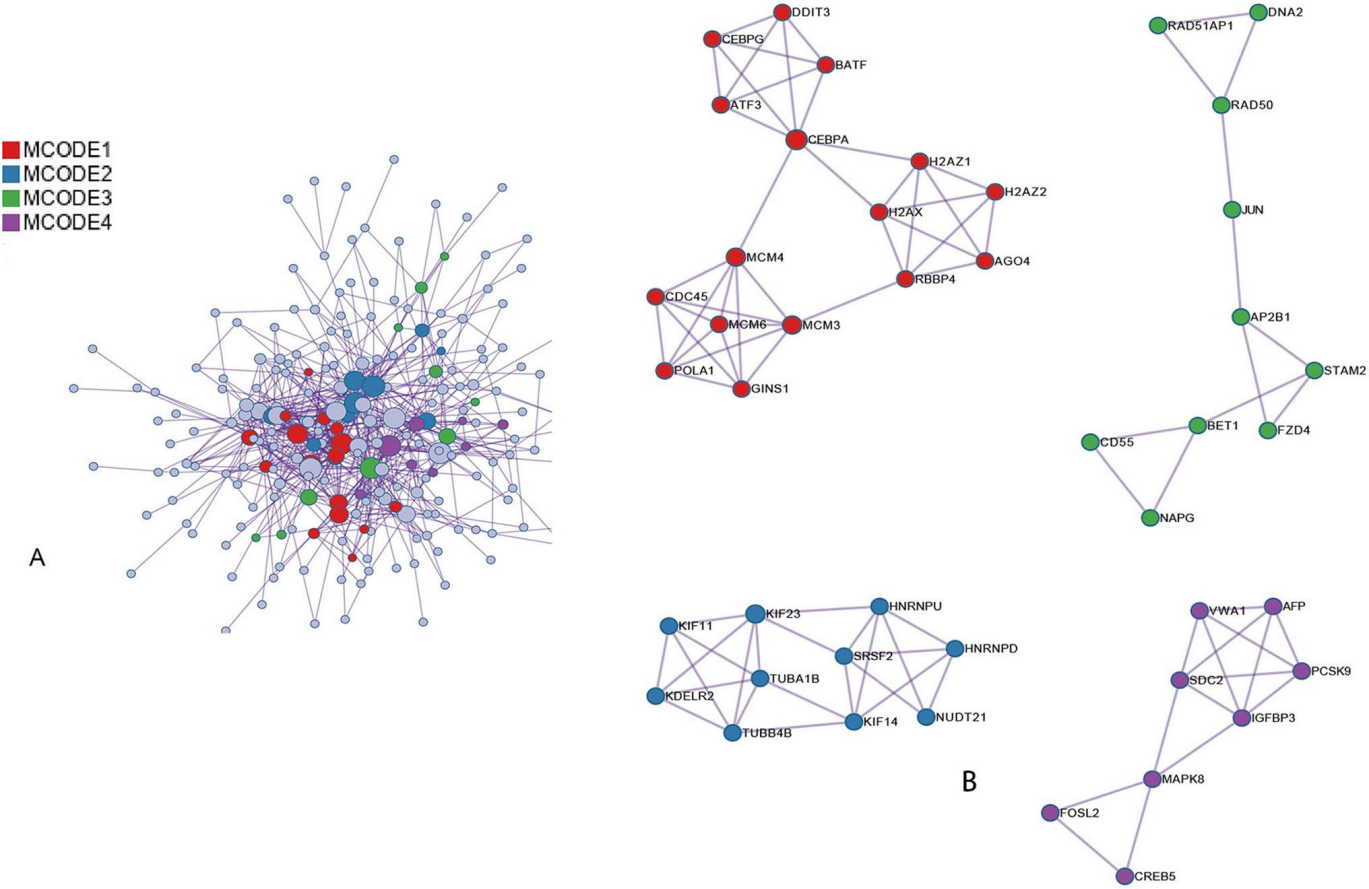
**A** PPI company network of the top 50 differential genes, **B** The key genes can be obtained from the mcode components of the top 50 genes

Metascape is a powerful software that can visually analyze various data. One of the important functions is PPI analysis. After analysis, we get relevant results (Fig. 3A). The MCODE networks identified for individual gene lists have been gathered and are shown in Fig. 3B. 44 hub genes were screened out. Differential genes mainly focus on cholesterol biosynthesis, tumor cell cycle, negative cell cycle regulation, cell stress response, DNA repair, liver development, G1/ s specific transcription, DNA-binding transcription factor activity regulation, idp53 downstream pathway, PID, ATF2 pathway, fissile cell response to astralization, tp53 transcriptional regulation and other pathways. Abnormal activity or dysregulation of these pathways play a key role in the occurrence and development of liver cancer, supporting the proliferation, invasion, anti-apoptotic ability of liver cancer cells and the expression of other cancer characteristics. Further study and analysis of the specific mechanisms of action of these pathways will contribute to a deeper understanding of the pathophysiological processes of liver cancer and is expected to provide important clues for the development of new therapeutic strategies.

### Immunoinfiltration analysis

**Fig. 4:**
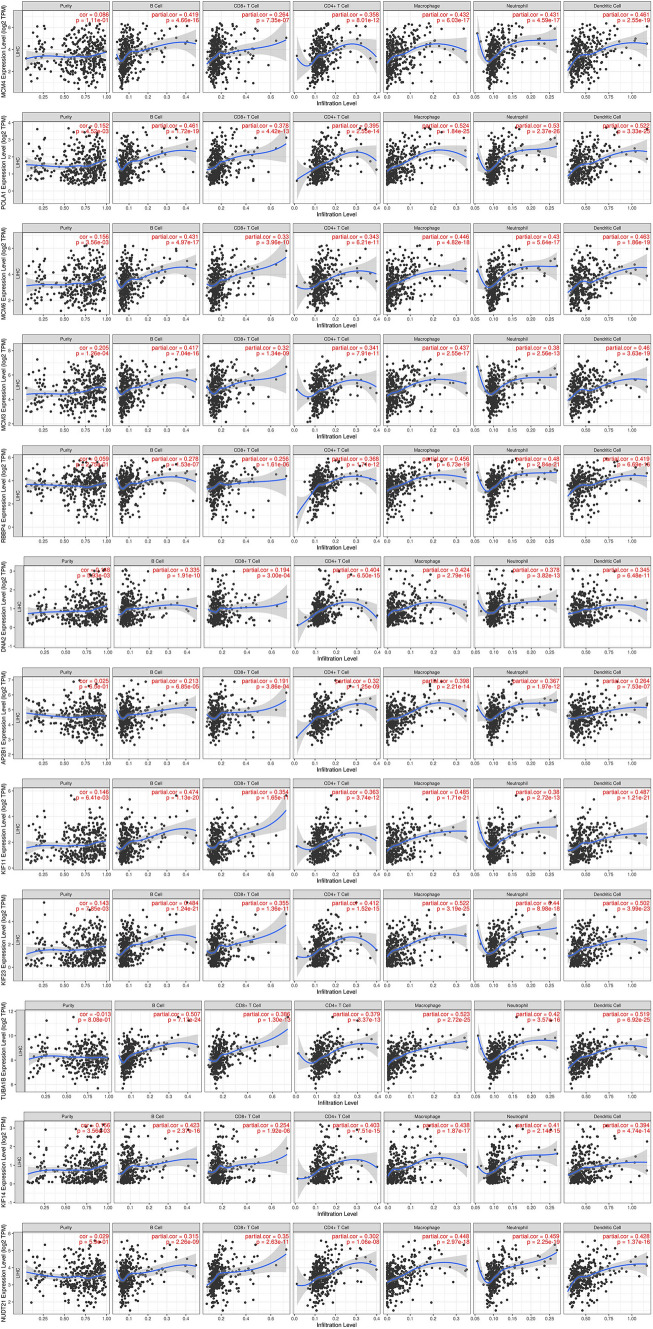
Immunoinfiltration analysis The 12 genes NUDT21, including MCM4, POLA1, MCM6, MCM3, RBBP4, DNA2, AP2B1, KIF11, KIF23, TUBA1B, KIF14 and MCM4, are closely correlated with tumor immune infiltration. These genes inhibited the proportion of B cells, CD8 + cells, CD4 + cells, macrophages, neutrophils, and dendritic cells in the immune potential environment of liver cancer, and the proportion of these cells decreased with the increase of gene expression (P < 0.05)

Research on the TIMER database found that twelve genes, MCM4, POLA1, MCM6, MCM3, RBBP4, DNA2, AP2B1, KIF11, KIF23, TUBA1B, KIF14 and NUDT21, are closely related to tumor immune infiltration (Fig. 4). These genes inhibited the proportion of B cell, CD8 + cell and CD4 + cell in the immune potential environment of liver cancer. These genes may play important roles in the regulation of tumor immune responses. For example, the MCM protein family (including MCM4, MCM6, and MCM3) plays a key role in DNA replication and cell cycle regulation and may affect tumor immune infiltration by affecting the proliferation and growth of tumor cells. POLA1 is a subunit of DNA polymerase α, involved in DNA synthesis, and its abnormal expression may affect the genetic stability and immunogenicity of tumor cells. Other genes such as RBBP4, DNA2, AP2B1, etc. may also affect tumor immune responses through their respective molecular functions.

### Analysis of hub gene in UALCAN

**Fig. 5:**
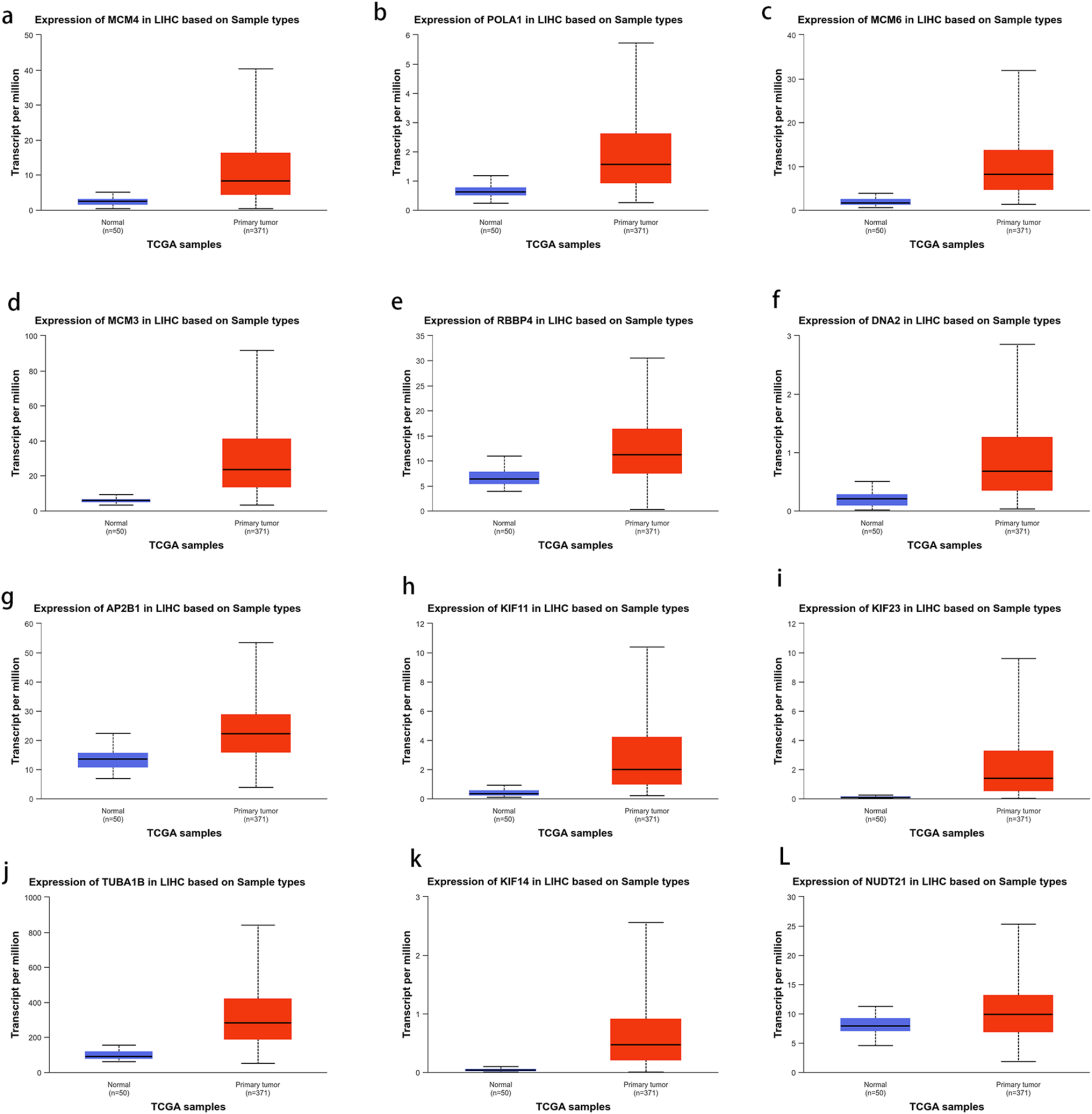
Expression difference and survival analysis of hub gene in hepatocellular carcinoma [MCM4 (**a**), POLA1 (**b**), MCM6 (**c**), MCM3 (**d**), RBBP4 (**e**), DNA2 (**f**), AP2B1 (**g**), KIF11 (**h**), KIF23 (**i**), TUBA1B (**j**), KIF14 (**k**), NUDT21 (**l**)]

**Fig. 6:**
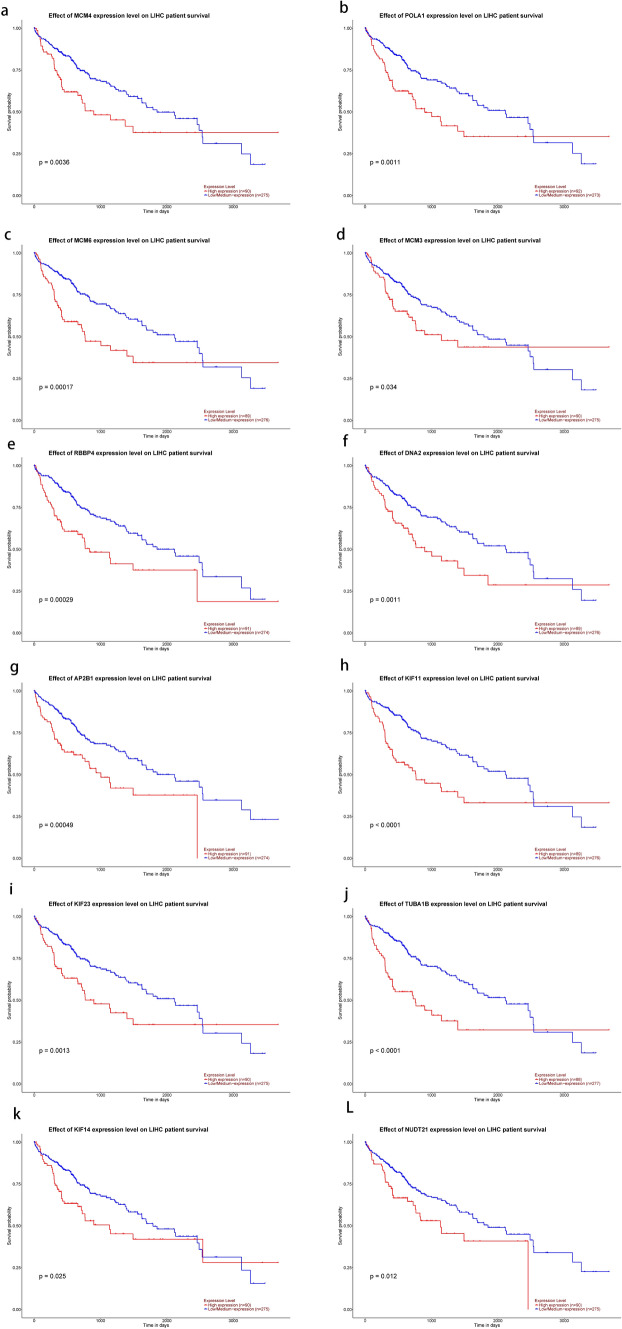
Expression difference and survival analysis of hub gene in hepatocellular carcinoma [MCM4 (**a**), POLA1 (**b**), MCM6 (**c**), MCM3 (**d**), RBBP4 (**e**), DNA2 (**f**), AP2B1 (**g**), KIF11 (**h**), KIF23 (**i**), TUBA1B (**j**), KIF14 (**k**), NUDT21 (**l**)]

**Fig. 7:**
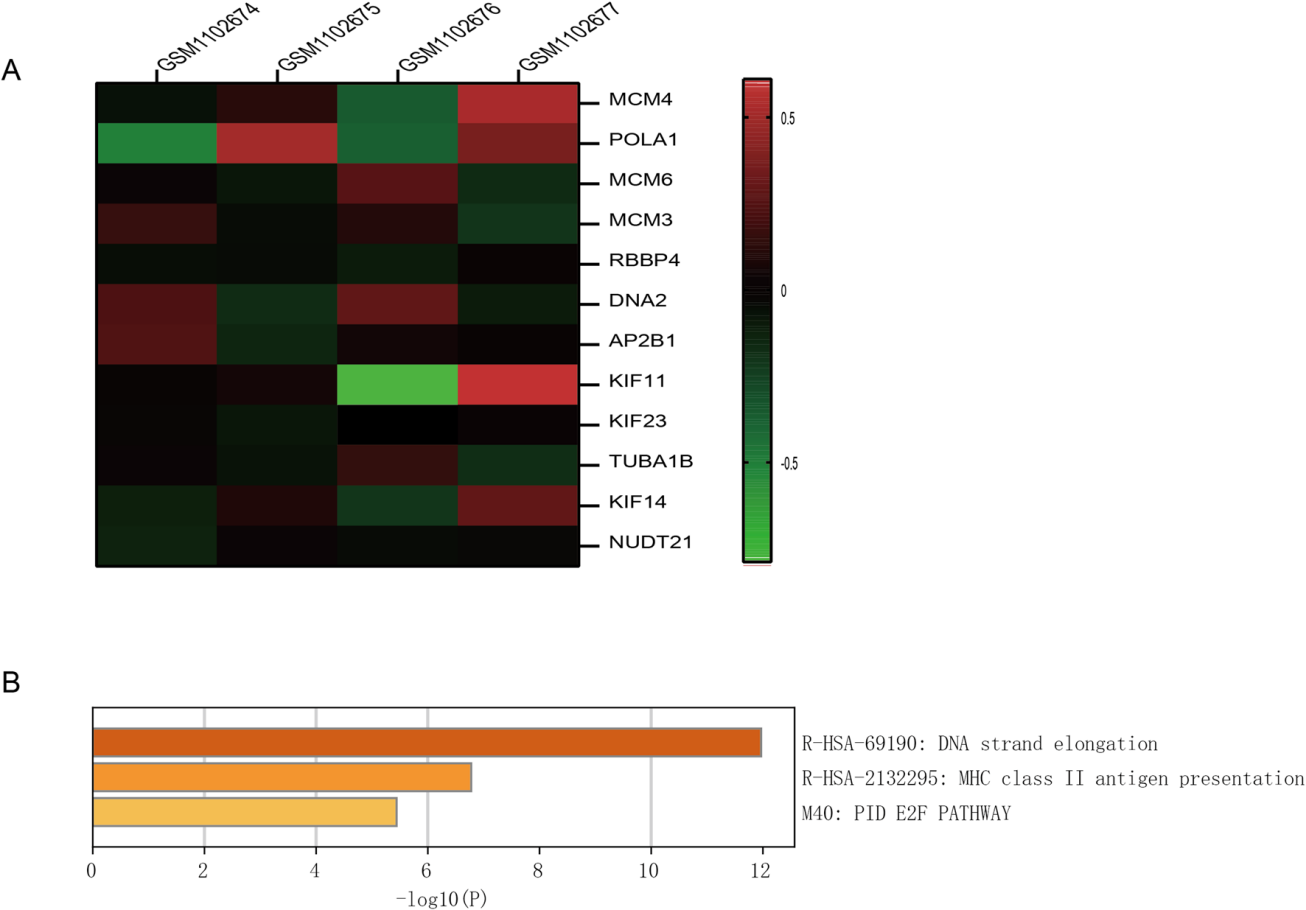
Enrichment analysis (**B**) and expression heat map (**A**) of 12 hub genes

The 44 genes were analyzed in UALCAN one by one, and the analysis showed that 12 genes were differentially expressed in HCC (MCM4 (Figs. 5a, 6a), POLA1 (Figs. 5b, 6b), MCM6 (Figs. 5c, 6c), MCM3 (Figs. 5d, 6d), RBBP4 (Figs. 5e, 6e), DNA2 (Figs. 5f, 6f), AP2B1 (Figs. 5g, 6g), KIF11 (Figs. 5h, 6h), KIF23 (Figs. 5i, 6i), TUBA1B (Figs. 5j, 6j), KIF14 (Figs. 5k, 6k), NUDT21 (Figs. 5l, 6l)), and their expression was closely related to the overall survival rate of HCC patients. The 12 genes were further enriched (Fig. 7B) and the expression heat map (Fig. 7A) was drawn. It is mainly enriched in E2F pathway and DNA replication pathway.

## Discussion

Sorafenib, lenvatinib and atezolizumab bevacizumab are FDA approved first-line drugs for the treatment of advanced liver cancer. At present, atezolizumab bevacizumab is the first line, sorafenib and lenvatinib are the second line. It is a multi-target kinase inhibitor of Raf kinase, vascular endothelial growth factor and platelet-derived growth factor receptor. Compared with the placebo group, sorafenib improves the survival time, and the median OS rate is 6.5 months [19]. However, patients with advanced HCC are mainly resistant to sorafenib, and their survival benefits are limited to 3–5 months, accompanied by serious side effects, which limit the clinical efficacy of sorafenib and greatly reduce the therapeutic effect of patients with advanced liver cancer [20]. Therefore, we need to find more effective methods. Celecoxib is a surprise, but its specific role in tumor needs further study, especially in hepatocellular carcinoma. The results of our bioanalysis showed that celecoxib combined with sorafenib could significantly inhibit the expression of hepatocellular carcinoma related genes, and provide help for predicting new targets for liver cancer therapy.

Another comparative study showed that celecoxib had the most significant anti-cancer effect compared to traditional NSaids. Harri’s model describes chemoprevention of celecoxib and Bloven in a rat model of breast cancer. The data showed that the incidence of tumor was reduced by 68% with celecoxib, 40% with ibuprofen, 86% with celecoxib, 52% with ibuprofen, followed by tumor volume, 81% with celecoxib and 57% with buprofen [21]. The antitumor effect of cyclooxygenase-2-derived prostaglandin E2 (PGE2) is based on a variety of mechanisms, including direct stimulation of cancer cell growth and neovascularization (neovascularization) [22, 23]. The best goal of chemotherapy is to prevent the growth of abnormal cells without any effect on the function of normal cells. Therefore, as a COX-2 inhibitor (celecoxib), it plays an important role in tumorigenesis. Its toxicity is lower than that of conventional non steroidal anti-inflammatory drugs [22], and its safety and efficacy parameters have been strictly determined for long-term treatment.

Our study further confirmed the shutdown gene and mechanism of celecoxib combined with sorafenib in the treatment of liver cancer. From our analysis results, the 12 genes (MCM4, POLA1, MCM6, MCM3, RBBP4, DNA2, AP2B1, KIF11, KIF23, TUBA1B, KIF14, NUDT21) in HCC tissues have significant expression differences, and also affect the overall survival rate of patients. These genes are mainly concentrated in DNA replication pathway, DNA replication stress should be regarded as a marker of cancer, because it may promote the development of cancer, and it is very common [24]. The results show that these genes are also enriched in E2F pathway, which is a pathway of many kinds of cancer, and is very important for the treatment of cancer. Cdk-rb-e2f pathway is very important for the regulation of cell proliferation. This suggests that celecoxib enhances the efficacy of tumor cells by regulating related genes to influence cell cycle pathways. Recently, studies have highlighted the additional role of this pathway, especially E2F transcription factor itself, in tumor progression, angiogenesis and metastasis. Specific E2Fs has prognostic value in breast cancer and has nothing to do with clinical parameters [25].

Immunoinfiltration analysis in tumor research focuses on characterizing the presence, composition, and function of immune cells in the tumor microenvironment (TME). The TIMER Database (Tumor Immune Estimation Resource) is a powerful tool that allows researchers to explore the interactions between somatic mutations, gene expression, and the abundance of various immune infiltrations in different cancer types. It was found that a group of 12 genes, mcm4, POLA1, MCM6, MCM3, RBBP4, DNA2, AP2B1, KIF11, KIF23, TUBA1B, KIF14 and nudt21, were correlated with tumor immune invasion. It suggests that there is a complex interaction between the basic cellular processes of tumor progression and the host immune response. These findings provide important clues for understanding the molecular mechanisms of tumor immune infiltration and provide potential targets for further research on tumor treatment and immunotherapy. Researchers can use the TIMER database to conduct further analysis of these genes, such as determining their expression patterns in different tumor types and stages, and their relationship with immune cells in the tumor microenvironment. This information can help reveal the mechanism of tumor immune evasion, guide the formulation of individualized treatment strategies, and improve the treatment effect and survival rate of tumor patients. Members of the MCM complex, such as MCM4, MCM6, and MCM3, are important components of the prereplication complex and play a role in licensing and regulating the initiation of DNA replication. Dysregulation of these proteins can lead to uncontrolled proliferation, which may affect immune recognition either directly by altering the antigenicity of tumor cells, or indirectly by producing a more aggressive phenotype that may attract or repel immune cells. As part of DNA polymerase alpha, POLA1 plays a critical role in DNA replication initiation. Abnormal POLA1 expression may affect genomic instability and the presentation of neoantigens, which are neoantigens produced by somatic mutations and can be recognized by the immune system. Impaired DNA synthesis mechanisms may also affect the overall fitness of tumor cells and their interactions with the immune system. RBBP4 is a regulatory subunit of several chromatin modification complexes, which may affect the transcriptional program of genes controlling immune response. The multifunctional nucleic acid helicase DNA2, which is involved in DNA repair and replication stress response, may influence how tumors evade immune surveillance. AP2B1, a component of clausin-coated pits, mediates endocytosis and may play a role in regulating immune cell receptor signaling or antigen uptake. KIF11, KIF23, and KIF14 are motor proteins involved in mitosis and intracellular transport that can influence tumor growth and spread, possibly affecting recruitment and localization of immune cells within TME. TUBA1B is a tubulin alpha chain that is a fundamental building block of microtubules and is essential for cell division and migration, including immune cell transport. NUDT21, a regulator of pre-mrna splicing, may influence the production of splicing variants that affect immune recognition, as alternative splicing is associated with the production of immunogenic peptides. Using the TIMER database to delve into the activity of these genes across multiple cancer types and stages can reveal patterns that predict patient outcomes, treatment response, or serve as biomarkers for immunotherapy. By elucidating the molecular mechanisms of immune infiltration in tumors, scientists can discover new targets to enhance immune cell infiltration, prevent immune escape, and develop personalized immunotherapy strategies to improve patient survival. Further experimental validation, such as functional analysis of patient samples and associated studies, will be necessary to confirm the specific role of these genes in tumor immune cell interactions.

This study was based on the GSE45340 dataset for analysis, and the sample size was relatively small, which may not fully represent the gene expression characteristics of all HCC patients. Therefore, the generalizability of the results may be limited. Only one public dataset (GSE45340) was used for analysis, and the lack of data verification from different research teams and experimental conditions may lead to insufficient stability and reliability of the results. Although potential key genes and signaling pathways were screened out through bioinformatics analysis, these findings are only based on gene expression data and lack further experimental verification. The specific molecular mechanisms need to be further explored through cell and animal experiments.

## Conclusion

Celecoxib combined with sorafenib is an effective method for the treatment of hepatocellular carcinoma. By detecting the expression levels of these genes, patients’ prognosis can be better assessed, and more effective treatment strategies can be developed to improve patient survival. Key genes such as MCM4, POLA1 and MCM6 are closely related to tumor immune invasion. Understanding the role of these genes in the tumor microenvironment can guide the selection and combination of immunotherapies and improve the effectiveness of immunotherapies.

## Data Availability

The data support the findings of this study are incorporated into the manuscript and available from the corresponding author upon reasonable request.

## Abbreviations

HCC: Hepatocellular carcinoma
GEO: Gene expression integrated database
TCGA: The cancer genome atlas
KEGG: Kyoto encyclopedia of genes and genomic pathways
GO: Gene ontology
DEGs: Differentially expressed genes
BP: Biological processes
MF: Molecular functions
CC: Cellular components
